# Psychological Distress, Resilience, and Immunoinflammatory Signatures in Healthcare Workers During COVID‐19

**DOI:** 10.1002/smi.70146

**Published:** 2026-02-10

**Authors:** Natália Gindri Fiorenza, Bruno Riccelli dos Santos Silva, Deniele Bezerra Lós, Nayana Holanda de Oliveira, Antônio Lucas Delerino, Débora Ferreira de Assis, Pedro Crosara Motta, Paulo Cesar Cortez, Maria Francilene Souza Silva, Marcela Helena Gambim Fonseca, João Alexandre Lobo Marques, Veridiana Pessoa Miyajima, Fábio Miyajima, Danielle S. Macedo

**Affiliations:** ^1^ Neuropsychopharmacology Laboratory, Drug Research and Development Center, Department of Physiology and Pharmacology Federal University of Ceara Fortaleza CE Brazil; ^2^ Life and Health Sciences Research Institute (ICVS) and ICVS/3B's ‐ PT Government Associate Laboratory Braga/Guimarães Portugal; ^3^ Computer Systems Engineering Laboratory, Department of Teleinformatics Engineering Federal University of Ceara Fortaleza CE Brazil; ^4^ Hematology and Hemotherapy Center of Ceara (HEMOCE) Fortaleza Ceara Brazil; ^5^ Ultrasound Laboratory, Department of Teleinformatics Engineering Federal University of Ceara Fortaleza CE Brazil; ^6^ Molecular and Epidemiological Analytical Laboratory Oswaldo Cruz Foundation (Fiocruz) Fortaleza Ceara Brazil; ^7^ Laboratory of Applied Neurosciences University of Saint Joseph Macao China

**Keywords:** COVID‐19, cytokines, healthcare workers, psychological distress, resilience

## Abstract

The COVID‐19 pandemic has profoundly affected healthcare workers, increasing vulnerability to neuropsychiatric disorders, such as anxiety and depression. Psychological distress may be shaped by resilience, coping behaviours, and immune dysregulation. We investigated psychological distress symptoms, resilience, alcohol use, and cytokine profiles in 1440 workers from four hospitals in Fortaleza, Brazil. Participants were classified as frontline or second‐line workers and assessed with the SRQ‐20, CD‐RISC, and AUDIT. Blood samples were analysed for SARS‐CoV‐2 antibodies and cytokines. Data were collected at two time points (August–October 2021; March–April 2022). Frontline workers reported higher distress, with decreased vital energy and somatic symptoms most prominent. Lower resilience scores correlated with all SRQ‐20 domains, while higher alcohol use was linked to decreased energy and depressive thoughts. Reduced anti‐spike antibody levels were also associated with greater distress. COVID‐19 infection and symptom severity were associated with more persistent mental distress symptoms. Sex‐specific immune signatures emerged: in women, lower interleukin (IL)‐7 and C‐X‐C motif chemokine ligand 9 (CXCL‐9) and higher IL‐27 correlated with depressive‐anxious mood and energy depletion; in men, IL‐18, IL‐9, and tumour necrosis factor beta (TNF‐β) were positively associated with distress. This study demonstrates that psychological distress among healthcare workers during COVID‐19 was shaped by resilience, alcohol use, infection severity, and sex‐dependent immune alterations. Strengthening resilience and targeting inflammatory pathways may help mitigate the long‐term mental health burden in this workforce during future public health crises.

## 1 Introduction

The COVID‐19 pandemic placed unprecedented psychological and occupational demands on healthcare workers, who faced high exposure to infection, increased workloads, and emotional strain. These stressors contributed to elevated rates of psychiatric disorders, including depression, anxiety, and psychological distress (Escorcia‐Del Chiaro et al. 2022; Lucas‐Hernández et al. 2022; Yang et al. 2023). Evidence from previous epidemics shows that frontline workers are particularly vulnerable to adverse mental health outcomes (Smith et al. 2017; Wadoo et al. 2020). During pandemics, health professionals frequently report stress, sadness, insomnia, emotional exhaustion, and fatigue, as well as persistent anxiety and cognitive difficulties (Amin et al. 2020; Chigwedere et al. 2021; Smith et al. 2017). Even those who do not initially present with psychological symptoms may develop problems later (Demartini et al. 2021).

During COVID‐19, frontline health professionals presented high prevalence rates of depression (50.4%), anxiety (44.6%), and insomnia (34%), with nurses and women being the most affected (Lai et al. 2020; Umbetkulova 2023; Elkholy et al. 2021). Furthermore, many patients infected with SARS‐CoV‐2 continue to experience long‐term sequelae known as post‐COVID conditions, which include fatigue, memory and concentration difficulties, depression, and anxiety (Kontoangelos et al. 2020; Soriano et al. 2022; Stefanou et al. 2022).

Biological mechanisms may help explain the persistence of psychological symptoms after COVID‐19. Immune hyperactivation during infection triggers the release of interferons (IFNs), interleukins (ILs), and other proinflammatory mediators (Sinha et al. 2020; Fajnzylber et al. 2020), which can disrupt blood–brain barrier integrity and promote neuroinflammation (Kempuraj et al. 2020; Roczkowsky et al. 2023). This process has been linked to COVID‐19–related neurological complications (Almutairi 2021; Rutkai et al. 2022; Ryu et al. 2024) and is also implicated in depression and anxiety (Adzic et al. 2018; Leighton et al. 2017). Cytokine alterations are increasingly explored as markers of stress‐related psychiatric disorders: reduced IL‐7 has been associated with major depression (Anjum et al. 2020); C‐X‐C motif chemokine ligand 9 (CXCL‐9), reflecting IFN‐γ activity, relates to depressive and anxiety symptoms during viral infections (Loftis et al. 2023; Rogers et al. 2020); and IL‐27, which has both pro‐ and anti‐inflammatory roles, has been linked to mood disturbances (Martinuzzi et al. 2021). However, the relevance of these cytokines as biomarkers of COVID‐19–related psychological distress remains unclear, particularly regarding potential sex‐specific patterns.

The pandemic has also amplified behavioural risk factors. Stressful experiences can increase alcohol consumption, a phenomenon termed stress‐induced alcohol seeking (Becker 2017; Heilig 2023; Iacoponi and de Jesus Mari 1989). Several studies reported heightened alcohol use during COVID‐19 lockdowns (Killgore et al. 2021; Murthy and Narasimha 2021). Alcohol consumption may temporarily relieve negative effects but is associated with a higher risk of psychiatric comorbidity, injuries, and premature mortality (Jones 2015).

In contrast, psychological resilience represents a dynamic process of adapting to adversity, trauma, or significant stressors (Fletcher and Sarkar 2013). During COVID‐19, reported lower resilience than other professionals; yet higher resilience is generally associated with better sleep, life satisfaction, and positive affect (Bozdağ and Ergün 2021). Evidence suggests that resilience and coping strategies can be enhanced through psychoeducational interventions, teamwork, and workplace support, which may mitigate vulnerability to stress (Bozdağ and Ergün 2021; Ménard et al. 2017).

Overall, these findings suggest that the psychological burden of health professionals during COVID‐19 is influenced by a complex interplay of occupational exposure, coping behaviours, resilience, and biological vulnerability. Despite high reported distress in this population, few studies have examined these psychosocial and biological factors together, particularly in low‐ and middle‐income countries where health systems face additional strain. In Brazil, high mortality, limited resources, and profound socioeconomic disruptions during the pandemic (Gama et al. 2023; Sott et al. 2022) further heightened the vulnerability of this workforce.

This study aimed to characterise the psychological distress experienced by health professionals during the COVID‐19 pandemic and to identify psychosocial and immunoinflammatory factors associated with this distress. Although numerous studies have documented elevated symptoms of anxiety, depression, and exhaustion in healthcare workers, few have jointly examined occupational exposure, resilience, coping behaviours, infection‐related variables, and cytokine profiles within the same population, particularly in low‐ and middle‐income settings. To address this gap, we pursued three objectives: (1) to evaluate whether frontline work, prior COVID‐19 infection, or illness severity were associated with specific domains of psychological distress; (2) to examine how resilience, alcohol use, and anti‐spike antibody levels related to these symptom domains; and (3) to investigate sex‐specific cytokine correlates of distress. We also applied exploratory factor analysis to determine whether SRQ‐20 domains reflect a unified latent distress construct and to evaluate whether occupational exposure or COVID‐19 infection contributes to this structure. Together, these analyses were designed to clarify modifiable psychosocial determinants and immunological signatures that may inform strategies to support the mental health of these workers during and after pandemic conditions.

## 2 Methods

### 2.1 Study Design and Participants

This multicenter cross‐sectional study was conducted in Fortaleza, Ceará, Brazil, with data collected at two recruitment periods using identical procedures: August–October 2021 and March–April 2022. All psychological, behavioural, and biological measures (SRQ‐20, CD‐RISC, AUDIT, antibody titres, and cytokines) were obtained contemporaneously within each period. Because the aim was to characterise overall patterns rather than temporal changes, data from both waves were pooled and analysed as a single cross‐sectional cohort.

Workers from multiple sectors were invited through institutional digital announcements. Eligible participants were adults (≥ 18 years) employed at the participating units who provided informed consent and completed the online survey. Institutional records did not allow for an exact estimate of the number of eligible workers; therefore, participation rates could not be calculated. Individuals with incomplete questionnaires were excluded.

Participants also provided blood samples at their workplaces. Based on professional role and exposure to COVID‐19 patients, workers were classified as frontline (e.g., nurses, nursing technicians, physicians involved in direct care) or second‐line (e.g., administrative staff, pharmacists, human resources personnel), following standardised criteria applied consistently across both recruitment periods (Bozdağ and Ergün 2021; Czepiel et al. 2024).

The study was approved by three Institutional Review Boards (approval numbers 50431921.9.0000.8152; 50431921.9.3001.5041; 50431921.9.3002.5044) and conducted in accordance with international ethical standards and Brazilian National Health Council Resolution No. 466/12. The dataset has not been previously analysed or published; any future work using specific subsamples (e.g., cytokines) will address distinct research questions.

### 2.2 Psychological and Behavioural Measures

Psychological distress was assessed using the Self‐Reporting Questionnaire (SRQ‐20), a 20‐item tool developed by the World Health Organisation to screen for mental‐emotional disorders, such as depression and anxiety (Harpham et al. 2003; Iacoponi and de Jesus Mari 1989; Santos et al. 2009). The items cover four domains: decreased vital energy, somatic symptoms, depressive‐anxious mood, and depressive thoughts. Total scores range from 0 to 20, with cutoffs of ≥ 7 for women and ≥ 5 for men used to define probable psychological distress (de Paula Guirado and Pereira 2016; Harpham et al. 2003; van der Westhuizen et al. 2016).

Resilience was measured using the Connor‐Davidson Resilience Scale (CD‐RISC‐10), a 10‐item questionnaire rated on a five‐point Likert scale. Higher scores reflect greater psychological resilience (Connor and Davidson 2003).

Alcohol use was evaluated with the Alcohol Use Disorders Identification Test (AUDIT), a 10‐item instrument designed to detect hazardous drinking and alcohol‐related problems. Higher scores indicate greater severity of alcohol‐related risk (Saunders et al. 1993).

In addition to these standardised measures, sociodemographic and clinical data, including age, sex, occupational role, and COVID‐19 infection history, were collected through the online survey. COVID‐19 infection history and disease severity were self‐reported and classified as asymptomatic, mild, moderate, or severe, following approaches commonly used in observational studies of healthcare workers during the pandemic (Lai et al. 2020).

### 2.3 Biological Measures

Blood samples were collected from participants at their workplaces using gel‐ and EDTA‐treated tubes. Serum and plasma were isolated by centrifugation at 3500 × g for 10 min, aliquoted, and stored at −80°C until analysis. Serum was subsequently sent to the Neuropsychopharmacology Laboratory at the University of Ceará for molecular analysis, and plasma samples were sent to the Serology Laboratory of the COVID‐19 Diagnosis Support Unit at the Oswaldo Cruz Foundation for antibody analysis.

All plasma samples were tested for IgG antibodies against the receptor‐binding domain of the SARS‐CoV‐2 spike protein using the Abbott SARS‐CoV‐2 IgG II Quant Assay. Antibody titres ≥ 50 AU/ml were considered positive.

To investigate immunoinflammatory correlates of psychological outcomes, a proportional subsample of 119 workers, stratified by sex and SRQ‐20 scores, was selected for cytokine analysis. Serum concentrations of 37 cytokines, chemokines, and growth factors were measured using the MILLIPLEX HCYTA‐60K‐37 Kit (Merck Ltd.). Data were acquired with the MAGPIX platform and analysed using Luminex 200 software. Median fluorescent intensity (MFI) values were used in all calculations, and quality was ensured with internal assay controls.

### 2.4 Statistical Analysis

All analyses were conducted using Python 3.12.3 (SciPy, Scikit‐posthocs) and JASP. Because data from two collection timepoints were pooled into a single cross‐sectional cohort, all analyses reflect contemporaneous associations rather than temporal effects.

#### 2.4.1 Data Screening and Assumption Checking

Data distribution and variance were evaluated using the Shapiro–Wilk and Levene tests. As most variables violated normality assumptions, non‐parametric statistical approaches were applied. Missing questionnaire data were minimal and addressed using listwise deletion for the corresponding analysis; cytokine values below detection limits were excluded according to manufacturer recommendations.

#### 2.4.2 Descriptive and Comparative Analyses

Results are reported as medians with interquartile ranges (IQR). Comparisons between two groups (e.g., frontline vs. second‐line workers) were performed using the Mann–Whitney *U* test, with effect sizes reported as rank‐biserial correlations. Multiple‐group comparisons (e.g., across COVID‐19 severity categories) were conducted using the Kruskal–Wallis test followed by Dunn's post hoc tests with Benjamini–Hochberg correction. Epsilon‐squared (ε^2^) was reported as an effect‐size estimate for Kruskal–Wallis tests.

#### 2.4.3 Correlation Analyses

Associations among cytokine levels, resilience (CD‐RISC), alcohol use (AUDIT), anti‐spike/RBD antibody titres, and SRQ‐20 domains were examined using Spearman's correlation. Exact *p*‐values and correlation coefficients (*ρ*) are presented. For visualisation, the matrix of correlation *p*‐values was imported into Morpheus (https://software.broadinstitute.org/morpheus/) and projected as a hierarchical, three‐dimensional heatmap.

#### 2.4.4 Sex‐Stratified Cytokine Analyses

Given strong prior evidence for sex differences in immune responses and vulnerability to stress‐related anxiety and depressive symptoms (Bangasser and Cuarenta 2021; Beery and Zucker 2011; Frohman Dafni et al. 2023) sex was pre‐specified as the primary stratification variable for cytokine analyses. Further stratification by age was not performed to prevent excessive subgrouping and loss of statistical power, particularly in the smaller male subsample. Age distributions are reported descriptively in Table 1.

**TABLE 1:** Sociodemographic and clinical data of the study group (*N* = 1440).

Characteristics of healthcare workers	*N* (%)
Sex, *n* (%)	
Men	313 (22%)
Women	1127 (78%)
Age (years), *n* (%)	
18–30	274 (19%)
31–50	685 (47%)
51–60	321 (23%)
≥ 60	160 (11%)
Race, *n* (%)	
White	377 (26.2%)
Black	50 (3.4%)
Mixed race	769 (53.6%)
Other	30 (2%)
Uninformed	214 (14.8%)
Working position, *n* (%)	
Frontline	628 (44%)
Second‐line	743 (52%)
Uninformed	69 (4%)
Had or had not COVID‐19	
Had COVID‐19	920 (64%)
Had not COVID‐19	520 (36%)
Disease severity, *n* (%)	
Asymptomatic	88 (9.5%)
Mild	505 (55%)
Moderate	302 (32.8%)
Severe	25 (2.7%)

*Note:* Disease severity was self‐reported and classified according to symptom‐based categories commonly adopted in observational studies of healthcare workers.

Abbreviation: Number of participants; %: Percentage of participants within the respective category.

#### 2.4.5 Exploratory Factor Analysis

To evaluate whether SRQ‐20 domains reflected a common latent structure of psychological distress and to assess whether occupational exposure or COVID‐19 infection contributed to this structure, an exploratory factor analysis was performed using a polychoric/tetrachoric correlation matrix, appropriate for ordinal variables (SRQ‐20 domains) and dichotomous indicators (frontline position and COVID‐19 infection). COVID‐19 infection was coded as 0 = no, 1 = yes; work position as 0 = second‐line, 1 = frontline.

Factor extraction followed Kaiser's criterion (eigenvalues > 1). Model adequacy was evaluated using χ^2^ tests and the Kaiser–Meyer–Olkin (KMO) measure. Factor loadings ≥ 0.40 were considered meaningful. The one‐factor solution was further supported by inspection of the scree plot and the conceptual coherence of the SRQ‐20 domains. The extracted latent factor (RC1), representing overall psychological distress, was then examined for shared variance with work position and COVID‐19 infection status.

Given the cross‐sectional design, all associations represent contemporaneous relationships and do not permit causal or directional inference; possible bidirectionality (e.g., distress influencing resilience or alcohol use, and vice versa) is acknowledged.

Given the exploratory nature of this study and the large population available for recruitment, no formal a priori power calculation was performed. However, the final sample of 1440 participants substantially exceeds commonly recommended thresholds for stable estimation of non‐parametric associations and exploratory factor models (MacCallum et al. 1999). For exploratory factor analysis, sample sizes above 300 are generally considered adequate (Lai et al. 2020). Therefore, the achieved sample provides sufficient statistical power for the planned analyses and supports the robustness of the reported results.

#### 2.4.6 Statistical Significance

**FIGURE 1:**
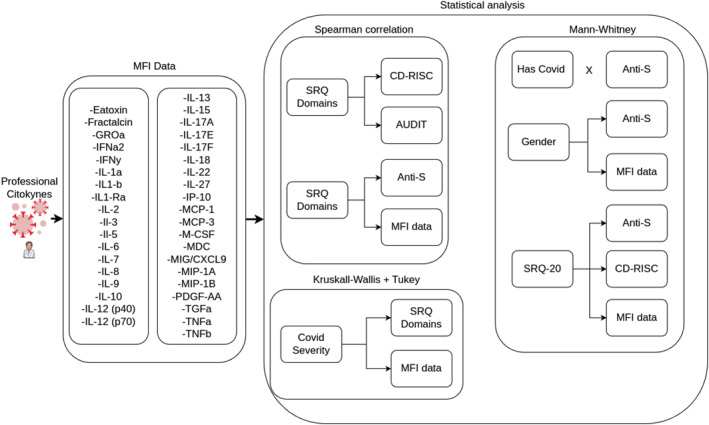
Workflow of statistical analysis. The statistical analysis was conducted using Python 3.12.3, utilising the SciPy and Scikit‐posthocs packages. The workflow began with data preprocessing and cleaning to ensure quality, followed by the computation of descriptive statistics to summarise key dataset characteristics. Inferential statistical tests were performed using SciPy, selecting parametric or non‐parametric methods based on data distribution. Given the non‐normal distribution, pairwise comparisons were conducted using non‐parametric tests. The figure outlines the sequential steps from data import and preprocessing to statistical testing and result interpretation.

Statistical significance was set at *p* ≤ 0.05 (two‐tailed). Effect sizes appropriate to each test are reported. Figure 1 presents a schematic summary of the analytic workflow.

## 3 Results

### 3.1 Demographic Characteristics

The study included 1440 healthcare workers, most of whom were women (78%). Age distribution was 18–30 years (19%), 31–50 years (47%), 51–60 years (23%), and ≥ 61 years (11%). Approximately 44% worked in frontline positions and 52% in second‐line roles. Most participants self‐identified as mixed race (53.6%), followed by White (26.2%) and Black (3.4%). A history of COVID‐19 infection was reported by 64% of the sample, with 55% experiencing mild and 32.8% moderate symptoms (Table 1). Participants who did not report their occupational category were excluded from position‐based analyses.

### 3.2 Work Position and Psychological Distress

**FIGURE 2:**
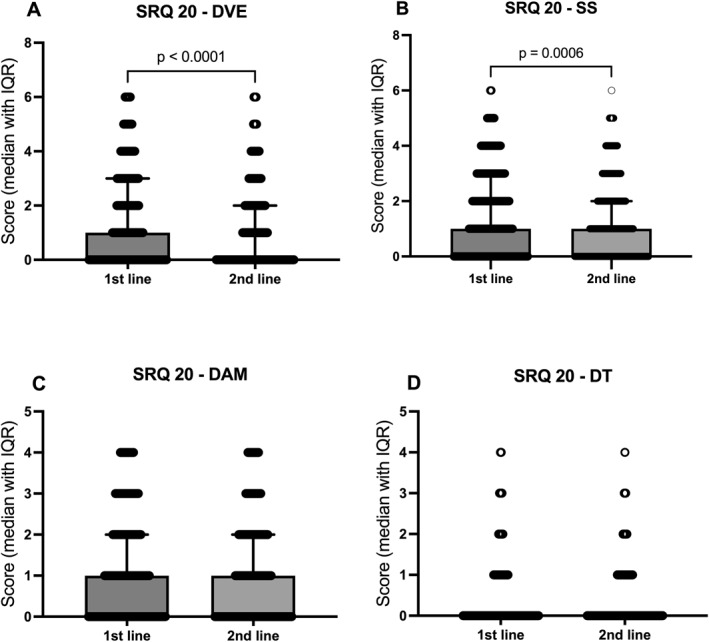
SRQ‐20 domain scores in frontline versus second‐line healthcare workers. SRQ‐20 domain scores are compared between Frontline (first line) and Second‐Line Healthcare Workers. The subdomains analysed include (A) Decreased Vital Energy (DVE), (B) Somatic Symptoms (SS), (C) Depressive‐Anxious Mood (DAM), and (D) Depressive Thoughts (DT), based on data from frontline HCWs (N = 628) and second‐line workers (N = 743). Data in the scatter dot plot are expressed as median with interquartile range (IQR) and were analysed using the Mann‐Whitney U‐test. Connecting lines indicate significant group comparisons (*p* ≤ 0.05), with exact *p*‐values displayed above each comparison.

Frontline workers reported higher decreased vital energy and somatic symptoms than second‐line workers (median differences = −0.40 and −0.29; 95% CIs [−0.57, −0.23] and [−0.44, −0.13], respectively; both *p* < 0.01; Figure 2A–B). No differences emerged for depressive‐anxious mood or depressive thoughts (Figure 2C–D).

**FIGURE 3:**
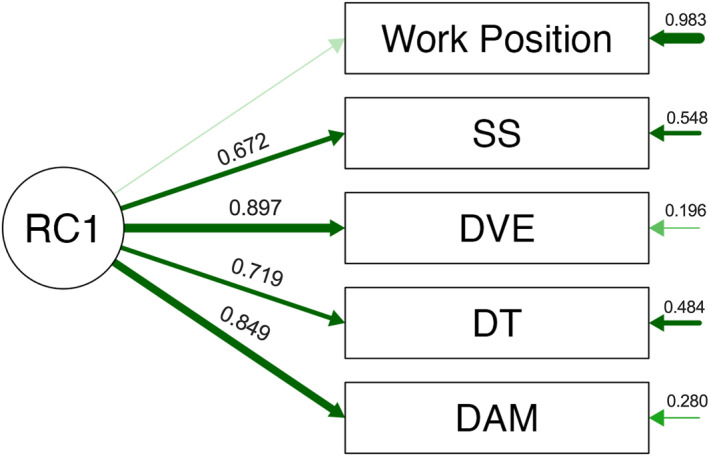
Exploratory factor model of SRQ‐20 psychological distress domains and work position. The exploratory factor model depicts the extracted latent factor RC1 and its standardised loadings onto the SRQ‐20 domains: Depressive Thoughts (DT), Decreased Vital Energy (DVE), Somatic Symptoms (SS), and Depressive–Anxious Mood (DAM), as well as work position (0 = second‐line; 1 = frontline). Values displayed on the arrows represent standardised factor loadings, and values shown to the right of each observed variable represent uniqueness (1 − communality). Arrow thickness reflects loading magnitude. Work position showed a negligible loading on RC1, indicating minimal shared variance with the latent psychological distress construct.

To evaluate whether psychological distress and occupational position reflected a shared latent structure, an exploratory factor analysis was conducted. Data showed excellent suitability (Bartlett's χ^2^(10) = 2831.69, *p* < 0.001; overall MSA = 0.80). A one‐factor solution (RC1) accounted for 50.2% of the variance (eigenvalue = 2.87) and captured a unified psychological distress dimension, with strong loadings for decreased vital energy (0.90), depressive‐anxious mood (0.85), depressive thoughts (0.72), and somatic symptoms (0.67). Work position did not load onto RC1 (0.02; uniqueness = 0.98), indicating no contribution to the latent construct (Figure 3).

### 3.3 Resilience, Antibody Production, Alcohol Consumption, and Cytokine Profiles

Resilience was negatively associated with all SRQ‐20 domains (*r* = −0.31 to −0.41, all *p* < 0.001), while higher anti‐spike/RBD antibody titres were associated with lower distress (*r* = −0.29 to −0.39, all *p* ≤ 0.001) (Table 2). Alcohol use showed small positive correlations with decreased vital energy (*r* = 0.10, *p* = 0.004) and depressive thoughts (*r* = 0.08, *p* = 0.012).

**TABLE 2:** Correlation between SRQ‐20 domains and AUDIT, CD‐RISC scales and Anti‐S/RBD antibody blood quantification.

	DVE	SS	DAM	DT
	Correlation coefficient (r); *p* value (*n* = 1440)			
CD‐RISC	−0.408; **≤ 0.0001**	−0.320; **≤ 0.0001**	−0.387; **≤ 0.0001**	−0.310; **≤ 0.0001**
AUDIT	0.098; **0.0036**	−0.0004; 0.989	0.0316; 0.353	0.084; **0.012**
ANTI‐S/RBD	−0.391; **≤ 0.0001**	−0.317; **0.0004**	−0.355; **≤ 0.0001**	−0.286; **0.001**

*Note:* Data were analysed by Spearman's correlation.

Abbreviations: Anti‐S/RBD, Anti‐Spike/RBD antibodies, a measure of the immune response to COVID‐19; AUDIT, Alcohol Use Disorders Identification Test, a tool for assessing alcohol consumption; CD‐RISC, Connor‐Davidson Resilience Scale, a measure of psychological resilience; DAM, Depressive‐Anxious Mood, a domain in SRQ‐20 evaluating mood‐related symptoms; DT, Depressive Thoughts, a domain in SRQ‐20 assessing the presence of depressive thoughts; DVE, Decreased Vital Energy, a domain in SRQ‐20 reflecting low‐energy levels; SRQ‐20, Self‐Reporting Questionnaire‐20; SS, Somatic Symptoms, a domain in SRQ‐20 covering physical manifestations of stress.

**FIGURE 4:**
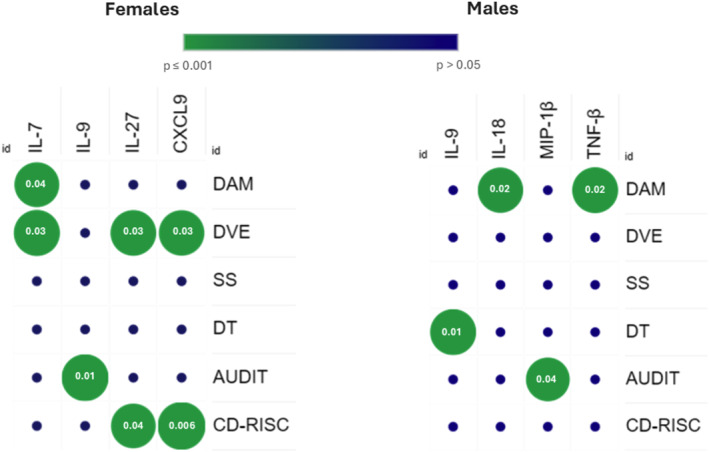
Sex‐stratified correlations between cytokines and psychological/behavioural measures. The plot summarises Spearman's rank correlations between serum cytokine concentrations and SRQ‐20 symptom domains, Decreased Vital Energy (DVE), Somatic Symptoms (SS), Depressive–Anxious Mood (DAM), and Depressive Thoughts (DT), as well as alcohol use (AUDIT) and resilience (CD‐RISC), shown separately for females (n = 100) and males (n = 19). Colour encodes statistical significance (green = lower *p*‐values; blue = non‐significant), and green circles denote associations meeting the nominal threshold of *p* ≤ 0.05, with the corresponding *p*‐values displayed inside the circles; non‐significant associations are shown as blue markers.

Sex‐stratified cytokine analyses revealed distinct patterns. Women: decreased vital energy correlated positively with IL‐27 (*r* = 0.21, *p* = 0.035) and negatively with IL‐7 (*r* = −0.22, *p* = 0.030). Depressive‐anxious mood correlated negatively with IL‐7 (*r* = −0.20, *p* = 0.046) and CXCL‐9 (*r* = −0.21, *p* = 0.038). Resilience correlated negatively with IL‐27 (*r* = −0.20, *p* = 0.048) and positively with CXCL‐9 (*r* = 0.27, *p* = 0.006). AUDIT scores correlated negatively with IL‐9 (*r* = −0.23, *p* = 0.019). Men: Depressive‐anxious mood correlated positively with IL‐18 (*r* = 0.50, *p* = 0.028) and TNF‐β (*r* = 0.51, *p* = 0.021). Depressive thoughts correlated positively with IL‐9 (*r* = 0.53, *p* = 0.019), and AUDIT with macrophage inflammatory protein‐1 beta (MIP‐1β) (*r* = 0.44, *p* = 0.049) (Figure 4).

### 3.4 COVID‐19 Symptom Severity and Psychological Outcomes

**FIGURE 5:**
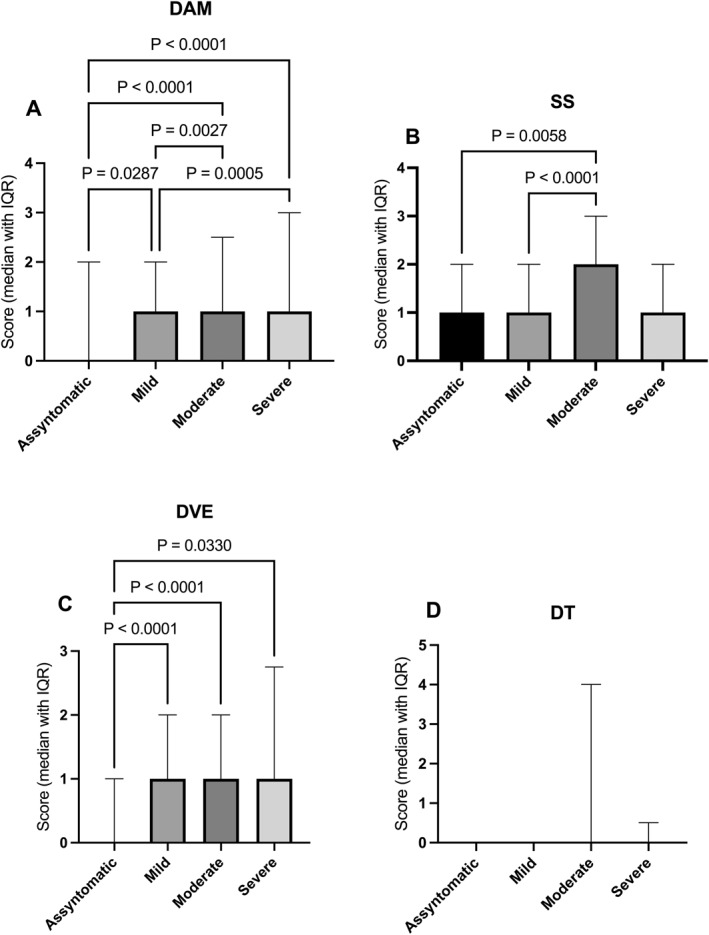
Effect of COVID‐19 severity on SRQ‐20 domain scores. Bars represent the median with interquartile range (IQR). Data from 920 participants were analysed using the Kruskal‐Wallis test, followed by Dunn's post hoc test. Connecting lines indicate significant group comparisons (*p* ≤ 0.05), with exact *p*‐values displayed above each line. DVE, Decreased Vital Energy; SS, Somatic Symptoms; DAM, Depressive‐Anxious Mood; DT, Depressive Thoughts.

Significant differences across severity groups were observed for depressive‐anxious mood (H(3) = 59.63, *p* < 0.001), with progressively higher scores from asymptomatic to severe cases (Figure 5A). Asymptomatic participants scored lower than mild, moderate, and severe groups (mean rank differences = −88.95 to −234.5, all *p* ≤ 0.0287), and mild cases scored lower than moderate and severe groups (mean rank differences = −111.6 and −145.5; both *p* ≤ 0.0027). Somatic symptoms scores also differed by severity (H(3) = 33.60, *p* < 0.001; Figure 5B); asymptomatic and mild participants showed lower somatic symptoms than moderate cases (mean rank differences = −102.9 and −104.5; *p* = 0.0058 and *p* < 0.0001). Decreased vital energy varied across severity groups (H(3) = 23.15, *p* < 0.001; Figure 5C). Asymptomatic participants scored lower than all symptomatic groups (mean rank differences = −127.5 to −152.3; all *p* ≤ 0.033). No severity‐related differences emerged for depressive thoughts (H(3) = 5.72, *p* = 0.126; Figure 5D).

### 3.5 Long‐Term Psychological Symptoms

**FIGURE 6:**
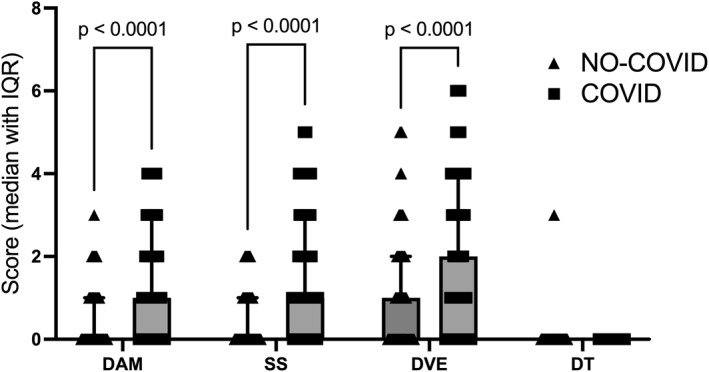
Effect of coronavirus infection on long‐term psychological symptoms. Scatter dot plot presenting the median with interquartile range (IQR) for psychological symptoms in participants with and without prior COVID‐19 infection (*N* = 245). Data were analysed using the Mann‐Whitney test with a two‐stage step‐up correction, considering the factors of COVID‐19 infection status (no‐COVID vs. COVID) and SRQ‐20 domains, including decreased vital energy (DVE), somatic symptoms (SS), depressive‐anxious mood (DAM), and depressive thoughts (DT). Connecting lines indicate significant group‐by‐group comparisons (*p* ≤ 0.05), with exact *p*‐values displayed above each line.

During the final study phase (March–April 2022), 245 workers reported infection status during the Omicron outbreak, of whom 160 (65%) had been infected two to three months earlier. Recently infected participants reported higher distress in depressive‐anxious mood (mean ranks = 129.1 vs. 84.94; *U* = 3475; *p* < 0.0001; *q* < 0.0001), somatic symptoms (mean ranks = 131.2 vs. 78.21; *U* = 2990; *p* < 0.0001; *q* < 0.0001), and decreased vital energy (mean ranks = 133.5 vs. 96.75; *U* = 4531; *p* < 0.0001; *q* < 0.0001). Depressive thoughts scores did not differ (*p* = 0.3764; *q* = 0.095) (Figure 6).

**FIGURE 7:**
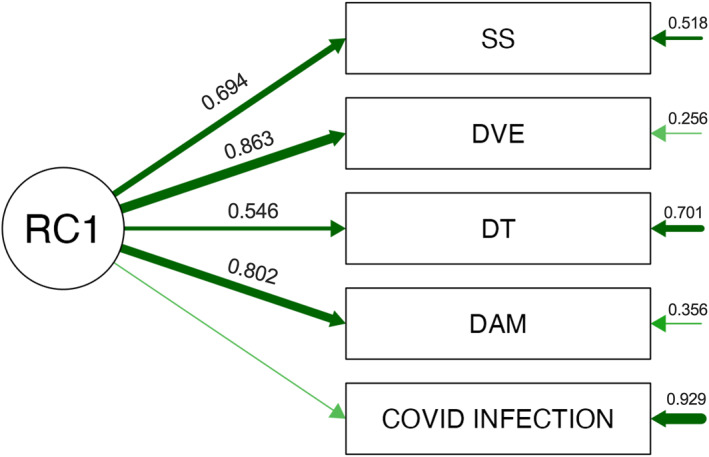
Exploratory factor model of SRQ‐20 psychological distress domains and COVID‐19 infection. The exploratory factor model depicts the extracted latent factor RC1 and its standardised loadings onto the SRQ‐20 domains—Depressive Thoughts (DT), Decreased Vital Energy (DVE), Somatic Symptoms (SS), and Depressive–Anxious Mood (DAM)—and COVID‐19 infection status (0 = no; 1 = yes). Values on arrows represent standardised factor loadings, and values shown to the right of each observed variable represent uniqueness (1 − communality). Arrow thickness reflects loading magnitude. COVID‐19 infection showed a negligible loading on RC1, indicating minimal shared variance with the latent psychological distress construct relative to the SRQ‐20 symptom domains.

A second exploratory factor analysis examined whether COVID‐19 infection contributed to the underlying distress structure. The one‐factor model demonstrated good fit (χ^2^(5) = 76.71, *p* < 0.001). RC1 captured the shared distress dimension, with loadings of 0.86 (decreased vital energy), 0.80 (depressive‐anxious mood), 0.69 (somatic symptoms), and 0.55 (depressive thoughts). COVID‐19 infection had a negligible loading (0.07; uniqueness = 0.93), indicating no contribution to the latent distress structure. RC1 accounted for 44.8% of the variance (eigenvalue = 2.24) (Figure 7).

## 4 Discussion

This study demonstrates a substantial psychological burden among healthcare workers during the COVID‐19 pandemic, shaped by occupational role, infection history, and illness severity. Consistent with prior evidence, frontline professionals reported higher somatic symptoms and greater reductions in vital energy than second‐line workers (Biber et al. 2022; Deng and Naslund 2020). However, exploratory factor analysis indicated that distress was primarily driven by individual symptom dimensions rather than work position itself, underscoring the multifactorial nature of psychological burden beyond categorical occupational exposure.

Among SRQ‐20 domains, decreased vital energy emerged as the most robust and consistent indicator of distress. This domain reflects physical and cognitive exhaustion, anhedonia, and persistent fatigue, symptoms particularly relevant in healthcare settings characterised by prolonged shifts, sustained vigilance, and extended use of personal protective equipment. Similar patterns have been reported in SRQ‐20 validation studies, in which reduced energy shows strong associations with functional impairment and occupational stress (Cavalcante‐Neto et al. 2016; de Paula Guirado and Pereira 2016; Iacoponi and de Jesus Mari 1989). Although the SRQ‐20 does not provide clinical diagnoses, its sensitivity to exhaustion‐related symptoms supports its utility for monitoring mental‐emotional burden in healthcare workers.

When we examined the latent structure of distress, the SRQ‐20 domains loaded strongly on a single factor (RC1), indicating a unified psychological distress dimension. Consistent with this factor structure, work position showed minimal alignment with RC1, reinforcing the distinction between symptom‐specific associations and global distress severity.

Resilience consistently emerged as a protective factor, with higher CD‐RISC scores associated with lower distress across all symptom domains. This finding aligns with evidence linking resilience to reduced risk of depression, anxiety, and burnout under chronic stress (Fletcher and Sarkar 2013; Serrão et al. 2021; Song et al. 2023). Importantly, resilience is dynamic and modifiable, highlighting its relevance as a target for organisational and psychosocial interventions (Edward 2005; Shrivastava and Desousa 2016).

Alcohol use showed modest associations with decreased vital energy and depressive thoughts, consistent with stress‐induced alcohol seeking models (Becker 2017; Heilig 2023). Increases in alcohol consumption during the pandemic have been widely reported (Killgore et al. 2021; Murthy and Narasimha 2021). Although effect sizes were small, these findings reinforce concerns regarding maladaptive coping behaviours in high‐stress occupational contexts.

### 4.1 Immunological Correlates of Psychological Distress

A novel contribution of this study is the association between reduced anti‐spike/RBD antibody titres and higher psychological distress scores, suggesting that sustained distress may compromise immune protection against SARS‐CoV‐2. Depressive symptoms have been shown to predict lower antibody titres after vaccination in healthcare workers (Kaneko and Tsuboi 2023)., although enhanced antibody responses have also been reported in previously infected individuals with depressive symptoms (Grignoli et al. 2023). These discrepancies likely reflect differences between acute and chronic stress exposure. Experimental and clinical data indicate that chronic stress impairs immunoglobulin production (Martínez‐Carrillo et al. 2011; Silberman et al. 2003), whereas acute stress may transiently enhance immune responses (Lone et al. 2022). Collectively, these findings support a model in which prolonged psychological distress undermines immune competence in healthcare professionals.

### 4.2 Sex‐Specific Cytokine Signatures

Women predominated in this cohort and were disproportionately affected by distress, consistent with global reports of higher depression, anxiety, and burnout among female healthcare workers during the pandemic (Biber et al. 2022; Czepiel et al. 2024; Huang et al. 2021). This vulnerability reflects a convergence of biological, occupational, and social factors, including hormonal influences, heightened frontline exposure, and unequal caregiving responsibilities (Dean et al. 2022; Dillon et al. 2022).

Sex differences in immune regulation and affective vulnerability provided a strong rationale for sex‐stratified cytokine analyses (Bangasser and Cuarenta 2021; Beery and Zucker 2011; Frohman Dafni et al. 2023; Raza et al. 2021). In women, IL‐7 and CXCL9 were negatively associated with depressive‐anxious mood and decreased vital energy, whereas IL‐27 showed positive associations with distress and inverse associations with resilience. IL‐7 plays a central role in lymphocyte homoeostasis and has been linked to depression in a sex‐dependent manner (Anjum et al. 2020; Hall et al. 2016). CXCL9, typically implicated in inflammatory depression phenotypes (Milenkovic et al. 2019), showed a protective association in women, suggesting context‐dependent immune–affective interactions. IL‐27, a pleiotropic cytokine, has also been increasingly implicated in mood disorders (Annam et al. 2024; Martinuzzi et al. 2021).

In men, depressive‐anxious mood correlated positively with IL‐18 and TNF‐β, while IL‐9 was associated with depressive thoughts and MIP‐1β with alcohol use. IL‐18 is a well‐established proinflammatory cytokine linked to depression and hypothalamic–pituitary–adrenal axis activation (Alcocer‐Gómez et al. 2014). Although TNF‐β and IL‐9 are less studied in psychiatric research, emerging evidence implicates both in neuroinflammatory and stress‐related processes (Cui et al. 2024).

### 4.3 Impact of COVID‐19 Infection Severity and Long‐Term Symptoms

COVID‐19 severity was strongly associated with psychological distress, with depressive‐anxious mood, somatic symptoms, and decreased vital energy increasing alongside illness severity. This pattern supports models proposing that viral infection exacerbates mental health outcomes through combined psychosocial and immunoinflammatory mechanisms (Kontoangelos et al. 2020; Soriano et al. 2022; Stefanou et al. 2022). Importantly, workers infected during the Omicron wave continued to report elevated distress months later, consistent with persistent neuropsychiatric symptoms described in post‐COVID conditions (Kobusiak‐Prokopowicz et al. 2022; Soriano et al. 2022). These findings indicate that long COVID contributes to sustained psychological distress risk among health professionals.

Consistent with the domain‐level findings, infection status showed negligible alignment with RC1 in the exploratory factor model, indicating limited shared variance with the overall latent distress dimension. This pattern suggests that infection‐related experiences may contribute to specific symptom clusters (particularly fatigue/somatic burden) rather than defining the core distress phenotype captured by SRQ‐20 domains.

These findings should be interpreted within the context of systemic challenges faced by Brazilian healthcare workers, including staffing shortages, sustained high exposure, and elevated mortality (Gama et al. 2023; Sott et al. 2022). Similar patterns have been observed internationally (Van Wert et al. 2022), although local socioeconomic and healthcare infrastructure factors likely shape vulnerability and resilience. While sex was prioritised for immunoinflammatory stratification, future studies with larger samples should examine age‐by‐sex interactions.

From a contextual perspective, these findings are consistent with the occupational realities observed in Brazilian healthcare settings during the pandemic. In our experience, many healthcare workers faced sustained exposure due to staffing shortages, prolonged shifts, and repeated redeployment across high‐risk units, often combined with multiple employment contracts across institutions. During early and peak phases of the pandemic, prolonged use of personal protective equipment, limited recovery time, and ongoing fear of infection were common and likely contributed to persistent fatigue, emotional exhaustion, and maladaptive coping behaviours. Although these contextual factors were not directly quantified in the present study, they provide a plausible occupational framework through which the observed patterns of decreased vital energy, distress persistence, and immune–behavioural associations can be interpreted.

### 4.4 Strengths, Limitations, and Future Directions

The cross‐sectional design precludes causal inference, and reverse causation cannot be excluded. Self‐report measures may introduce reporting bias, and the smaller cytokine subsample, particularly among men, limits the precision of sex‐specific findings. The single‐region design may also constrain generalisability.

Key strengths include the large multicenter cohort spanning distinct pandemic phases and the integration of psychological, behavioural, epidemiological, and immunological data. The use of validated instruments, standardised laboratory methods, and appropriate non‐parametric analyses enhances robustness. Identification of sex‐specific immune signatures provides testable hypotheses for future longitudinal and mechanistic studies.

## 5 Conclusions

Psychological distress among healthcare workers during COVID‐19 reflects a complex interaction between occupational demands, infection‐related factors, resilience, behavioural responses, and sex‐dependent immune alterations. Although causal pathways cannot be established, the findings suggest that interventions targeting resilience, adaptive coping, and immune‐related vulnerability may help mitigate long‐term mental health consequences in this population. Longitudinal studies are needed to clarify temporal dynamics and biological mechanisms linking stress, immunity, and mental health in healthcare workers.

## Author Contributions

D.S.M. and F.M.: conceived the idea and got the research funding. N.G.F., N.H.O., D.B.L., V.P.M. and M.H.G.F.: performed the data collection. N.G.F., D.B.L., V.P.M. and M.H.G.: performed the laboratory experiments. B.R.S.S., A.L.D., D.F.A., P.C.M., P.C.C., J.A.L.M.: data curation and statistical analyses. D.S.M. and N.G.F.: wrote the first draft. All authors approved the final version of the manuscript.

## Funding

Brazilian Governmental Institutions Ceará Foundation for Support to Scientific and Technological Development (FUNCAP) and Fundação Oswaldo Cruz (FIOCRUZ) Grant number: FIO‐0167‐00065.01.00/20 SPU N°: 06531047/2020.

## Ethics Statement

The study was conducted in accordance with the ethical standards of the Declaration of Helsinki and Brazilian regulations (National Health Council Resolution No. 466/2012). Written informed consent was obtained from all participants. The research protocol was approved by the ethics committees of Hospital Geral Dr. César Cals (CAAE 50431921.9.3001.5041), Hospital São José (CAAE 50431921.9.3002.5044), and the Haematology and Hemotherapy Center of Ceará (CAAE 50431921.9.0000.8152).

## Conflicts of Interest

The authors declare no conflicts of interest.

## Use of AI

During the preparation of this work, we used CHATGPT 4o to improve readability. After using this tool/service, the authors reviewed and edited the content as needed and take full responsibility for the content of the published article.

## Supporting information


Supporting Information S1

Supporting Information S2

## Data Availability

Raw data is available upon request to the corresponding author.
