# Supplementary material for: Psychological Distress, Resilience, and Immunoinflammatory Signatures in Healthcare Workers During COVID‐19
Source: Stress Health. 2026 Feb 10;42(1):e70146. doi: 10.1002/smi.70146 (PMC12891298; doi:10.1002/smi.70146)
Supplement: Supplementary file 1 — Supporting Information S1 [file SMI-42-e70146-s001.docx]

**Supplementary File 1 — Self-applicable Questionnaire of Mental Health for Health Workers**

| **Self-applicable Questionnaire of Mental Health for Health Workers** |
| --- |
| **Project:** Impact and consequences of COVID-19 on health workers in the state of Ceará: An integrated clinical-psychiatric and molecular assessment of workers' health |

| *INFORMED CONSENT FORM (ICF)*  Dear participant: You are invinting to participate in the research called IMPACT AND CONSEQUENCES OF COVID-19 ON HEALTH WORKERS IN THE STATE OF CEARÁ: AN INTEGRATED CLINICAL-PSYCHIATRIC AND MOLECULAR ASSESSMENT OF WORKERS' HEALTH.  The aim of this survey instrument is to assess the development or worsening of mental disorders, resulting from the COVID-19 pandemic, in frontline and second-line health workers so that we can devise strategies for managing the mental health of these workers and coping with future epidemics.  This is a voluntary survey and you have the right not to take part or to withdraw at any time. If you wish, you will have all the information you want, before, during and after the research. We also guarantee that your personal data will not be disclosed and will remain anonymous.  The questionnaire will be stored and can be used in future research, for which anonymity and confidentiality will also be guaranteed. If you decide to take part in this study, you will receive, via a messaging app, a link to a form which you will have to fill in and send back online, free of charge. The form contains questions about your mental health, vaccination, alcohol consumption, quality of life and well-being. The answers you give us will be used exclusively for the scientific purposes of this study and will take an average of 15 minutes to complete.  By filling it out, you will be helping us to understand how the COVID-19 pandemic, as well as exposure to SARS-CoV-2, has impacted on the mental health of professionals who are on the first and second front lines of the COVID-19 response.  Any questions regarding the research can be answered by the researchers.  I declare for all intents and purposes that I have read the information contained in this document, have been duly informed about the procedures that will be carried out, have understood the objectives, risks and benefits of participating in the research and accept the invitation to participate.*****   1. YES 2. NO |
| --- |

| 1. *Identification* |
| --- |
| A.1 Full name  A.2 Birthdate  A.3 Sex   1. Male 2. Female   A.4 Hospital/Health Center  A.5 Working position  A.6 Race   1. White 2. Black 3. Mixed race 4. Other   A.7 Have you already answered this questionnaire after taking the first dose of coronavirus vaccine?   1. YES 2. NO |

| B. Inquérito soro-epidemiológico e vacinal |
| --- |
| B.1 Have you had the first dose of coronavirus vaccine?   1. YES 2. NO   B.2 Have you had the second dose of coronavirus vaccine?   1. YES 2. NO   B.3 Have you had COVID-19 ??   1. Yes, but I haven't sought medical attention. 2. Yes, I sought medical assistance but no tests were ordered. 3. Yes, I sought medical assistance and underwent imaging tests (X-ray, CT, etc.) 4. Yes, I sought medical assistance and underwent laboratory tests (serology, swab, etc.) 5. I did not have COVID-19.   B.4 How you consider your ilness was?   1. Mild 2. Moderate 3. Severe 4. Assyntomatic (without symptoms)   B.5 If you take medical appoitament, Where is it?   1. Basic Health Unit - UBS (health post) 2. Basic Health Unit - UBS (health post) 3. Family Health Program - PSF 4. Field Hospital 5. SUS Hospital 6. Private Health Network 7. I didn't seek medical assistance   B.6 Have you been POSITIVELY diagnosed with COVID-19 through any LABORATORY EXAMS?   1. YES 2. NO   B.7 Which?   1. Nasal Swab (PCR) 2. Quick test 3. Sorological test (IgG/IgM)   B.8 Did you use some medicine to prevention or theatment of COVID-19?   1. YES 2. NO   B.9  Which?   1. Azithromycin 2. Oseltamivir (Tamiflu) 3. Hydroxychloroquine/Chloroquine 4. Ivermectin 5. Corticoids (beta/dexamethasone/prednisone/etc). 6. Zinc 7. Vitamin D 8. Nitazoxanide (Anitta) 9. Anti-inflammatories 10. Antipyretics 11. Other   B.10 If you used other medicine, please indicate its name above:  B.11 When you took the FIRST DOSE of coronavirus vaccine?  B.12 Which laboratory produced this vaccine?   1. Sinovac (Coronavac)/  Butantan 2. BioNTech/Fosun Pharma/Pfizer (BNT162) 3. Universidade de Oxford/AstraZeneca (AZD1222)/ Fiocruz 4. Bharat Biotech (Covaxin) 5. I don’t know.   B.13 Did you have any of the reactions below when you received the FIRST dose of the vaccine? (highlight all symptoms that you had)   1. Pain in application site 2. Fever 3. Headache 4. Runny nose 5. Cough 6. Body pain 7. Fadigue 8. Other symptoms 9. I have not symptoms   B.14 Please, describe other symptoms that you had:  B.15 When you took the SECOND DOSE of the vaccine?  B.16 Which laboratory produced this vaccine?   1. Sinovac (Coronavac)/  Butantan 2. BioNTech/Fosun Pharma/Pfizer (BNT162) 3. Universidade de Oxford/AstraZeneca (AZD1222)/ Fiocruz 4. Bharat Biotech (Covaxin) 5. I don’t know.   B.17 Did you have any of the reactions below when you received the FIRST dose of the vaccine? (highlight all symptoms that you had)   1. Pain in application site 2. Fever 3. Headache 4. Runny nose 5. Cough 6. Body pain 7. Fadigue 8. Other symptoms 9. I have not symptoms   B.18 Please, describe other symptoms that you had:  B.19 Do you believe that the vaccine you have taken will immunize you from COVID19?   1. YES 2. NO |

| *C. Self Reporting Questionnaire (SRQ-20) (Please, you must considering the last 30 days to answer)* |
| --- |
| C.1 Do you often have headaches?   1. YES 2. NO     C.2 Is your appetite poor?   1. YES 2. NO     C.3 Do you sleep badly?   1. YES 2. NO   C.4 Are you easily frightened?   1. YES 2. NO     C.5 Do your hands shake?   1. YES 2. NO     C.6 Do you feel nervous tense or worried?   1. YES 2. NO     C.7 Is your digestion poor?   1. YES 2. NO     C.8 Do you have trouble thinking clearly?   1. YES 2. NO   C.9 Do you feel unhappy?   1. YES 2. NO   C.10 Do you cry more than usual?   1. YES 2. NO   C.11 Do you find it difficult to enjoy your daily activities?   1. YES 2. NO   C.12 Do you find it difficult to make decisions?   1. YES 2. NO   C.13 Is your daily work suffering?   1. YES 2. NO   C.14 Are you unable to play a useful part in life?   1. YES 2. NO   C.15 Have you lost interest in things?   1. YES 2. NO   C.16 Do you feel that you are a worthless person?   1. YES 2. NO   C.17 Has the thought of ending your life been in your mind?   1. YES 2. NO   C.18 Do you feel tired all the time?   1. YES 2. NO   C.19 Do you have uncomfortable feelings in your stomach?   1. YES 2. NO   C.20 Are you easily tired?   1. YES 2. NO |

| D. *The Alcohol Use Disorders Identification Test (AUDIT) (Please, you must considering the last 12 months to answer)* |
| --- |
| D.1 How often do you have a drink containing alcohol?  0 = Never  1 = Monthly or less  2 = 2-4 times a month  3 = 2-3 times a week  4 = 4 or more times a week  D.2 How many standard drinks containing alcohol do you have on a typical day when drinking?  0 = 1 or 2  1 = 3 or 4  2 = 5 or 6  3 = 7 to 9  4 = 10 or more  D.3 How often do you have six or more drinks on one occasion?  0 = Never  1 = Less than monthly  2 = Monthly  3 = Weekly  4 = Daily or almost daily  D.4 During the past year, how often have you found that you were not able to stop drinking once you had started?  0 = Never  1 = Less than monthly  2 = Monthly  3 = Weekly  4 = Daily or almost daily  D.5 During the past year, how often have you failed to do what was normally expected of you because of drinking?  0 = Never  1 = Less than monthly  2 = Monthly  3 = Weekly  4 = Daily or almost daily  D.6 During the past year, how often have you needed a drink in the morning to get yourself going after a heavy drinking session?  0 = Never  1 = Less than monthly  2 = Monthly  3 = Weekly  4 = Daily or almost daily  D.7 During the past year, how often have you had a feeling of guilt or remorse after drinking?  0 = Never  1 = Less than monthly  2 = Monthly  3 = Weekly  4 = Daily or almost daily  D.8 During the past year, have you been unable to remember what happened the night before because you had been drinking?  0 = Never  1 = Less than monthly  2 = Monthly  3 = Weekly  4 = Daily or almost daily  D.9 Have you or someone else been injured as a result of your drinking?  0 = No  1 = Yes, but not in the past year  2 = Yes, during the past year  D.10 Has a relative or friend, doctor or other health worker been concerned about your drinking or suggested you cut down?  0 = No  1 = Yes, but not in the past year  2 = Yes, during the past year |

| *E. Connor-Davidson Resilience Scale (CD-RISC) (Please, you must considering the last 30 days to answer).* |
| --- |
| E.1 Able to adapt when changes occur.  0 = Not true at all  1 = Rarely true  2 = Sometimes true  3 = Often true  4 = True nearly all the time  E.2 Can deal with whatever come.  0 = Not true at all  1 = Rarely true  2 = Sometimes true  3 = Often true  4 = True nearly all the time  E.3 Try to see humorous side of things.  0 = Not true at all  1 = Rarely true  2 = Sometimes true  3 = Often true  4 = True nearly all the time  E.4 Stress can make me stronger.  0 = Not true at all  1 = Rarely true  2 = Sometimes true  3 = Often true  4 = True nearly all the time  E.5 Tend to bounce back after illness or hardship.  0 = Not true at all  1 = Rarely true  2 = Sometimes true  3 = Often true  4 = True nearly all the time  E.6 Can achieve my goals, even if I have obstacles.  0 = Not true at all  1 = Rarely true  2 = Sometimes true  3 = Often true  4 = True nearly all the time  E.7 Under pressure, I stay focused.  0 = Not true at all  1 = Rarely true  2 = Sometimes true  3 = Often true  4 = True nearly all the time  E.8 Not easily discouraged by failure.  0 = Not true at all  1 = Rarely true  2 = Sometimes true  3 = Often true  4 = True nearly all the time  E.9 Strong even when dealing with challenges.  0 = Not true at all  1 = Rarely true  2 = Sometimes true  3 = Often true  4 = True nearly all the time  E.10 Able to handle unpleasant or painful feelings  0 = Not true at all  1 = Rarely true  2 = Sometimes true  3 = Often true  4 = True nearly all the time |
