# Supplementary material for: Psychological Distress, Resilience, and Immunoinflammatory Signatures in Healthcare Workers During COVID‐19
Source: Stress Health. 2026 Feb 10;42(1):e70146. doi: 10.1002/smi.70146 (PMC12891298; doi:10.1002/smi.70146)
Supplement: Supplementary file 2 — Supporting Information S2 [file SMI-42-e70146-s002.docx]

**Supplementary File 2**

Table S1 – Analyses of the coronavirus infection severity and proinflammatory cytokine release for Chi square Kruskal-Wallis with Tukey's multiple comparison test.

| # | Mdif | Lower X upper | | p |
| --- | --- | --- | --- | --- |
| Interferon gama (IFN-y) | | | | |
| Assyntomatic X Mild | -62.6488 | | -99.441 X -25.856 | 0.0001 |
| Assyntomatic X Moderate | -57.894 | | -95.095 X -20.692 | 0.0006 |
| Assyntomatic X Severe | -44.975 | | -86.318 X -3.631 | 0.0275 |
| IL-18 | | | | |
| Mild X Severe | 159.3943 | | 25.694 X 293.094 | 0.0128 |
| TNF-β | | | | |
| Mild X Severe Frontline | 109.7017 | | 22.305 X 197.098 | 0.0078 |
| Moderate X Severe | 100.8993 | | 11.363 X 190.435 | 0.0208 |

Table S2 – Summary of the data present in the study results section

| Fig. # | Mean | S.E.M | N | Statistical method used |
| --- | --- | --- | --- | --- |
| **2 – Work position and SRQ-20 domains** | | | | |
| Decreased vital energy | | | | Mann-Whitney U-test |
| Frontline | 1.513 | 0.069 | 628 |  |
| 2^nd^ line | 1.113 | 0.054 | 743 |  |
| Somatic symptom | | | |  |
| Frontline | 1.548 | 0.060 | 628 |  |
| 2^nd^ line | 1.260 | 0.051 | 743 |  |
| Depressive and anxiety mood | | | |  |
| Frontline | 1.202 | 0.051 | 628 |  |
| 2^nd^ line | 1.090 | 0.045 | 743 |  |
| Depressive thoughts | | | |  |
| Frontline | 0.356 | 0.030 | 628 |  |
| 2^nd^ line | 0.296 | 0.023 | 743 |  |

Table S2 continued

| **Fig. #** | r | I.C | p | Statistical method used |
| --- | --- | --- | --- | --- |
| **3A – Correlaction in women** | | | |  |
| Decreased vital energy |  |  |  | Spearman’s correlation |
| IL-7 | - 0.217 | - 0.402, - 0.016 | 0.030 |  |
| Il-27 | 0.211 | 0.010, 0.396 | 0.035 |  |
| CXCL-9 | - 0.207 | - 0.393, - 0.006 | 0.038 |  |
| Depressive and anxiety mood | | | |  |
| IL-7 | - 0.200 | - 0.386, 0.007 | 0.046 |  |
| AUDIT | | | |  |
| IL-9 | - 0.234 | - 0.417, - 0.033 | 0.019 |  |
| CD-RISC |  |  |  |  |
| Il-27 | - 0.198 | - 0.384, 0.002 | 0.048 |  |
| CXCL-9 | 0.272 | 0.072, 0.451 | 0.006 |  |
| **3B – Correlaction in men** | | | |  |
| Depressive and anxiety mood | | | | Spearman’s correlation |
| IL-18 | 0.502 | 0.004, 0.801 | 0.028 |  |
| TNF-β | 0.511 | 0.031, 0.800 | 0.021 |  |
| Depressive thoughts | | | |  |
| IL-9 | 0.530 | 0.036, 0.816 | 0.019 |  |
| AUDIT | | | |  |
| MIP-1β | 0.444 | -0.041, 0.761 | 0.049 |  |

Table S2 continued

| Fig. # | Mean | S.E.M | N | Statistical method used |
| --- | --- | --- | --- | --- |
| **4 –** **Disease severity and SRQ-20 domain** | | | | |
| Depressive and anxiety mood | | | | Kruskal-Wallis test |
| Assyntomatic | 0.917 | 0.051 | 520 |  |
| Mild | 1.121 | 0.054 | 505 |  |
| Moderate | 1.434 | 0.060 | 505 |  |
| Severe | 1.525 | 0.080 | 301 |  |
| Somatic symptoms | | | |  |
| Assyntomatic | 1.341 | 0.148 | 520 |  |
| Mild | 1.343 | 0.064 | 505 |  |
| Moderate | 1.947 | 0.092 | 505 |  |
| Severe | 1.520 | 0.361 | 301 |  |
| Decreased vital energy | | | |  |
| Assyntomatic | 0.977 | 0.146 | 520 |  |
| Mild | 1.390 | 0.074 | 505 |  |
| Moderate | 1.728 | 0.104 | 505 |  |
| Severe | 1.560 | 0.342 | 301 |  |
| Depressive thoughts | | | |  |
| Assyntomatic | 0.227 | 0.057 | 520 |  |
| Mild | 0.340 | 0.031 | 505 |  |
| Moderate | 0.455 | 0.049 | 505 |  |
| Severe | 0.320 | 0.125 | 301 |  |
| **5 – SRQ-20 domain after 2-3 months COVID infection** | | | | |
| Decreased vital energy | | | | Kruskal-Wallis test |
| Without COVID | 1.349 | 0.185 | 86 |  |
| With COVID | 2.119 | 0.149 | 159 |  |
| Depressive and anxiety mood | | | |  |
| Without COVID | 1.035 | 0.138 | 86 |  |
| With COVID | 1.478 | 0.109 | 159 |  |
| Somatic symptoms | | | |  |
| Without COVID | 1.140 | 0.161 | 86 |  |
| With COVID | 1.704 | 0.119 | 159 |  |
| Depressive thoughts | | | |  |
| Without COVID | 0.337 | 0.078 | 86 |  |
| With COVID | 0.465 | 0.068 | 159 |  |
